# Professionalism in medical education: the state of the art

**DOI:** 10.5116/ijme.6626.583a

**Published:** 2024-04-29

**Authors:** Alexandra M. Goodwin, Scott W. Oliver, Isla McInnes, Kirsty F. Millar, Kathleen Collins, Catherine Paton

**Affiliations:** 1 Medical education department, NHS Lanarkshire, Bothwell, UK

**Keywords:** Professionalism, medical education, clinicians, Professional identity

## Introduction

The topic of professionalism in healthcare is becoming increasingly prominent in the literature and in practice. Various definitions have been proposed;^1^^,^^2^ we consider ‘professionalism’ to encompass the behaviours, attributes and characteristics that complement technical training and elements of ‘being an employee’, and which collectively enable the practice of healthcare by an individual or group of practitioners.

The importance of professionalism in healthcare cannot be understated. Clinicians are consistently rated amongst the most trusted members of society, with patients’ perceptions of ‘professionalism’ key to their relationship with healthcare practitioners.^3^ Across the world, the majority of actions taken by healthcare regulators against individual practitioners relate to professional conduct rather than technical error. Recruitment activity for junior and senior healthcare practitioners tends to focus upon professionalism domains to select and differentiate candidates, particularly for more competitive and/or senior posts.

In this context, it is surprising that most professionalism training across the healthcare professions resides within the hidden curriculum. In the past decade many formal undergraduate and postgraduate curricula have taken steps to address this, but training in these vast and complex domains remains scarce in comparison to education around technical skills. Similarly, while there is an expanding literature describing professionalism domains, interventions and pedagogy, there is a conspicuous lack of conference activity focused on this area. Specifically, most major medical education events in recent memory include sessions or individual speakers with a title relevant to professionalism, but there are few, if any, events dedicated to the study of professionalism amongst healthcare professionals.

“Professionalism in Healthcare: Adapting to the Seasons” was an international conference convened to address this need. Whilst originally planned as a face-to-face event in May 2020, the pandemic necessitated a postponed online event that was held on Friday 1st October 2021 via an online platform (Zoom). The aims of the meeting were to share knowledge and expert opinion around professionalism in practice, its associated pedagogy, and to delineate key areas for future research.  Key themes arising from the meeting included the complex nature of culture and how it influences professionalism identity formation; aspects of professionalism in postgraduate practice; how we might positively influence professionalism through better understanding of how it is learnt and taught, and the ways in which we might improve staff wellbeing in challenging healthcare contexts. Many speakers considered their topic through the lens of undergraduate medical education, but across the event a broad range of views were heard to include undergraduate and postgraduate medicine, nursing, allied health and social care professionals. In this commentary we describe the ‘state of the art’ of professionalism in medical education as discussed at the meeting and call for targeted work to develop our understanding and practice in this broad and evolving field.

### Impact of culture on professionalism

The impact of culture on individual and team performance is well recognised, and the conference provided a platform to articulate how this creates conflict for learners and teachers. Speakers discussed prejudicial behaviours at societal and organisational level and how these can spill over into university and clinical environments. Evidence was presented that at least some students witness or personally encounter prejudicial behaviour and/or language during their clinical training. Barriers to addressing this were reported to include clinical and academic hierarchies, difficulty challenging these hierarchies, and uncertainty about addressing difficult behaviour by patients and colleagues. Several groups had implemented ‘active bystander’ training, intending to equip students with skills to challenge these behaviours.^4^ Citing positive participant feedback, speakers recommended such programmes to other healthcare educators. A model for de-escalating challenging behaviours in the intensive care clinical environment was also presented.

Specific challenges were raised in relation to the broadening socio-demographic of medical school entrants. Conflict related to diversity was described as being overt, for example, racist language; or covert, for example, financial hardship that constrained full participation in student life. A further challenge requiring to be addressed was dissonance between the manner in which medical undergraduates are taught to treat patients, and their own lived experience as students.^5^ This was compellingly described in a qualitative study where graduate entry medical students were invited to reflect on triumphs and challenges on their teaching and assessment of professionalism in medical school.^6^ The professional values students were expected to model, versus those encountered in practice during their time on clinical attachments, were often at odds with one another. The authors described a need for specific practical skills to enable students to “speak up” when encountering these conflicts.

### Professional identity formation at medical school

Medical students’ transition through the early years of medical school was seen to influence their perception of what their future professional identity should be.^7^ A ‘cultural indoctrination’ was described, where students were moulded to be accepting of traditional hierarchies and to compete with peers for clinical experience. The resulting emotional burden on students was described alongside faculty interventions to support students. Paradoxically, these support initiatives were described as contributing further to student stress and potentially worsening students’ experience overall, particularly where they included compulsory extra sessions, or extra coursework such as pieces of reflecting writing.

Negative elements of academic culture and clinical learning environments were also described. Data was presented to suggest that some students had learnt to develop a “thick skin” in order to survive harassment or bullying behaviour;^7^ indeed, this maladaptive behaviour was sometimes viewed positively by senior faculty. This was contrasted with the Canadian Medical Education Directives for Specialists (CanMEDS) framework ideal whereby physicians work collegially and collaborate with colleagues and patients without significant power gradients being evident.^2^

### Postgraduate professional practice

Most professionalism work to date has concerned undergraduate health professionals, but conference also heard data regarding various studies and interventions related to postgraduate professional practice. One survey demonstrated factors that could disadvantage locum doctors in comparison with substantive members of staff. These included; a lack of formal induction, limited/absent educational and pastoral support, and perceptions by peers and other colleagues that they must be incompetent to not have a substantive role. The authors suggested further work to correct these issues and explore further links with recruitment and attainment, and clinical outcomes. In a keynote address delegates were presented with an analysis of ‘seven steps to patient safety’,^8^ emphasising the dynamic nature of safety (and professionalism), and the need for realism when contemplating ideals versus realities of day-to-day working in practice.^9^

### Pedagogy of professionalism

Conference acknowledged the increasing emphasis given by undergraduate healthcare curricula to professionalism, however the optimal means of learning, teaching and assessment remain subject to debate. Several groups described professionalism tutorials seeking to introduce regulatory guidance and frameworks to undergraduate students. The presented data suggested that combinations of didactic teaching and case study discussion could be used to introduce such material. The importance of interactive group discussion was highlighted both for teaching purposes, but also to provide insight to faculty regarding individual students’ perceptions and working understanding of professionalism in practice. Speakers also presented data suggesting that attendance at such sessions enabled students to more reliably and confidently implement reflective practice and other aspects of professionalism immediately following participation.

Others reported interventions to enhance the teaching of specific elements of professionalism. A befriending scheme which matched third year medical students with “longer stay” hospital inpatients was reported to have improved students communication skills, but also enabled students to understand patients in their broader life context. This information was gathered via online reflective log forms completed by the students. There were also anecdotal positive benefits reported for patients in terms of their mood, well-being and a reduction in reported loneliness.

Online learning and its potential benefits for teaching professional behaviours was described in the context of allied health professionals. One study concerned students studying physiotherapy, nursing, oral health or social work. The authors suggested a flipped classroom^10^ approach optimised online teaching about professional skills such as data governance, and professional behaviours such as working in teams and regulatory codes of conduct. Online learning was particularly relevant in the context of the Covid19 pandemic, and also the anecdotally reported logistical challenges in organising teaching events for increasing class sizes amongst healthcare courses.^11^ A further presentation considered how undergraduate medical students might safely navigate online social media. An e-learning module was developed, intending to highlight potential pitfalls and to provide basic strategies for mitigating risks.

The conference also considered more traditional clinical skills training, and the professionalism challenges these can bring. The terminology of ‘pimping’ was used to contextualise different teaching styles, including where individual students were singled out or otherwise ‘questioned to humiliate’.^7^ In addition to the immediate, negative, interpersonal impact of this teaching style, data was presented to suggesting these reinforced negative hierarchical structures in healthcare. It was suggested that faculty guidance regarding acceptable questioning style could counteract this practice and hopefully, thus, these negative effects.

Delegates noted the tendency for medical schools to regard male gender as being the ‘normal’ default. This was highlighted by observation that medical students tended to lack training in basic practical issues when examining patients with breasts.^12^ This could extend to other aspects of intimate examination, and potentially also to other protected characteristics.^13^ It was recommended that medical schools made efforts to teach students about gender differences, and to ensure that assessment strategies supported the practice of clinical skills using all genders of patients.

Constraints and opportunities provided by the Covid19 pandemic were actively discussed. The normalisation of online learning was acknowledged but also that in the face of adversity many clinical teams had come together with a new sense of collegiality. Seeking to capturing this sense of community, one medical school had developed ‘Operation Colleague’: a project embedding medical students into clinical teams.^14^ This utilised a traditional clinical apprenticeship model to optimise clinical capacity to host student attachments whilst observing social distancing and clinical workload constraints in the midst of the pandemic.

Conference recognised the potentially emotive responses triggered by many elements of professionalism. One speaker considered this through the lens of undergraduate medical students’ mental health difficulties.^15^ Strategies to actively maintain learner wellbeing when teaching sensitive subjects included providing self-care instruction and signposting contextually appropriate resources within learning materials. For example, actively teaching students about sleep hygiene, diet and exercise measures; and publishing relevant charity factsheets and contact details for university counselling services during teaching sessions about depression or suicidal ideation. Feedback data was presented suggesting these additional resources enhanced student experience and reduced barriers to tackling sensitive topics.

A professionalism objective structured clinical examination (OSCE) station implemented was described. The scenarios were based on regulatory guidance that had been taught through a series of workshops earlier in the course. Students who had participated in the workshops and the OSCE had subsequently reflected on their changing perspectives of what professionalism meant to them in practice. This generated various areas of future work including the development of additional training materials to improve future student cohorts’ preparation for entering postgraduate clinical practice.

### Wellbeing of healthcare professional staff and students

In the context of the Covid19 pandemic, and the extensively documented challenges this posed to healthcare professionals, the final session of the conference was themed to explore aspects of staff wellbeing.

Some speakers tackled basic physiological considerations, for example the opening keynote speaker reviewed the physiology of sleep and its relationship with health and cognition. A further speaker described challenges posed by menstruation for staff working in clinical environments. Practical means by which employers could provide support for this were also articulated during the session. These included the provision of free menstrual products, reorganisation of break times and bathroom proximity to working areas.

Other speakers explored psychological elements of workplace stress and wellbeing for clinicians. ‘Resilience training for foundation doctors’ was described in a study which aimed to explicitly teach the nature of stress and burnout to its participants.^16^ Coping strategies were introduced for specific predictable scenarios including when mistakes were made in the clinical workplace. Another study described a Balint group debriefing intervention used to explore students’ understanding of the role played by emotion in their relationships with patients.^17^ Qualitative feedback suggested students found this a useful opportunity, but more data was needed before the authors could firmly determine its value within the workplace or curriculum. A further study described an unpublished randomised controlled trial comparing an intensive, mindfulness-based, intervention with a weekly group discussion of recently encountered professionalism issues during a clinical attachment. Early results suggested a possible role for the mindfulness intervention, but again the authors recommended a larger scale investigation to inform final conclusions. Similar work has been published in the context of postgraduate education, suggesting its feasibility and hinting at efficacy, however further study is required to draw any firm conclusions.^18^

In postgraduate practice the conference also heard of a system used to support staff in a residential childcare setting. This drew on a described model for supporting staff who were routinely exposed to traumatic situations in the course of their work.^19^ The ‘whole system’ approach was reported

to have improved workplace team dynamics, reduced work-associated trauma and lessened the risk of burnout.

There was general agreement between all speakers that some form of formal timetabled wellbeing activities were required in undergraduate and postgraduate practice, and that further work should address the optimal nature, timing and resource allocation to make this a success.

## Conclusions

The conference brought together a diverse range of academic and clinical staff from around the world, representing almost every healthcare profession and discipline. Speakers reported a vast array of work in the professionalism field aiming to educate, support and empower colleagues to provide the best possible care for patients. The positive role of professionalism was striking throughout every formal presentation, panel discussion, and question and answer session. Anecdotally, professionalism in the healthcare context can be confined to regulatory documents and ‘avoiding being unprofessional’; Professionalism in Healthcare: Adapting to the Seasons clearly articulated professionalism as a means of bringing colleagues together, as a mechanism to optimise care, and to enable colleagues to “thrive, not just survive”.

This meeting report provides a comprehensive overview of the state of professionalism in healthcare at the time of the conference. We commend our delegates and speakers for their energy, effort and enthusiasm to enhance professional practice across the healthcare world. We recommend that the issues and challenges described herein form the basis for future research in this area, and hope the interventions described can be implemented at local levels across healthcare settings. Moreover, we emphasise the need to promote professionalism in medical education between professional disciplines, across national borders and throughout the world.

Since the event we have maintained and developed an evolving range of professionalism teaching resources that are hosted on our website. Encouraged by the significant, worldwide positive engagement with this conference, we plan future similar events, and we look forward to providing further commentary on global progress in due course.

### Conflicts of Interest

The authors declare they have no conflicts of interest.
